# Boosted NH_3_ Selective Catalytic Oxidation Activity over V-Pt-Ti Catalysts: Insight into Preparation Method Effects

**DOI:** 10.3390/ma19010194

**Published:** 2026-01-05

**Authors:** Yu Gao, Lipeng Wang, Kun Li, Yongbo Ji

**Affiliations:** China Waterborne Transport Research Institute, Beijing 100088, China; gaoyu@wti.ac.cn (Y.G.);

**Keywords:** V-Pt-Ti catalysts, selective catalytic oxidation of NH_3_, preparation methods, precipitation

## Abstract

In this work, V-Pt-Ti catalysts were synthesized employing impregnation (IP), precipitation (PC), sol-gel (SG), thermal decomposition (TD), and hydrothermal (HD) methods. A systematic study has been carried out to investigate impacts of various preparation methods on the performance of NH_3_ selective catalytic oxidation (SCO) at temperatures from 150 °C to 450 °C. N_2_ adsorption/desorption, XPS, XRD, H_2_-TPR, NH_3_-TPD, O_2_-TPD, SEM, TEM, and in situ DRIFTS were adopted to characterize the physico-chemical property of V-Pt-Ti catalysts. The results suggested that V-Pt-Ti catalysts synthesized by precipitation methods (denoted as VPT-PC) exhibited notably better SCO performance across the 150–450 °C temperature range compared with those produced by impregnation (IP), sol-gel (SG), thermal decomposition (TD), and hydrothermal (HD) methods. The outstanding performance of the VPT-PC catalyst could be ascribed to its larger surface area, higher relative contents of Pt^0^, V^5+^, and O_α_, more abundant surface acid sites, and better redox property. In situ DRIFTS results suggested that NO_2_ species could participate in NH_3_ oxidation reaction on the surface of the VPT-PC catalyst, which was beneficial for improving the SCO activity.

## 1. Introduction

Global shipping industry is gradually adopting NH_3_-fueled engines as main propulsion devices for newly built large cargo ships [1,2,3] to meet increasingly stringent GHG emission reduction requirements. NH_3_-fueled engines carry an unavoidable tendency to emit NH_3_, which puts natural environment at risk [4,5,6,7,8]. It is essential to develop feasible and high-performance methods to remove NH_3_ from exhaust gas emitted by NH_3_-fueled engines. In this regard, selective catalytic oxidation (SCO) is regarded as a more promising method in comparison to other approaches [9,10,11] due to its ability to efficiently convert NH_3_ to N_2_ under conditions of comparatively high temperatures and space velocities.

The catalyst is the core factor that determines the effectiveness of SCO technology. Throughout the last several decades, a range of SCO catalyst types have been created, falling into two main categories: noble metal catalysts and transition metal oxide catalysts. Noble metal catalysts usually possess strong oxidative catalytic activity toward NH_3_, which gives them superior low-temperature NH_3_ conversion capabilities [11,12,13]. However, they are prone to over-oxidizing NH_3_ into NO_x_, leading to comparatively low N_2_ selectivity [13,14,15]. In contrast, the transition metal oxide catalysts exhibit mild performance in catalytic oxidation of NH_3_, thereby showing high N_2_ selectivity, but their low-temperature NH_3_ conversion capabilities are deficient [15,16,17].

To develop SCO catalysts with a broad temperature window and excellent N_2_ selectivity, an approach to combining transition metal oxides with small-amount noble metals is proposed [18,19,20,21]. This approach leverages the internal selective catalytic reduction (i-SCR) mechanism: NH_3_ is first oxidized to NO by O_2_ under the action of noble metals; then, NO reacts with NH_3_ in the SCR reaction under the action of transition metal oxides. 

In the past few years, Pt-V bimetallic catalysts designed via the aforementioned approach have attracted intensive attention owing to their simultaneous achievement of excellent NH_3_ conversion performance and N_2_ selectivity at low temperatures. Byun et al. synthesized a Pt_0.3_V_3_W_2_/TiO_2_ catalyst with a Pt content of 0.3 wt.%. This catalyst achieved a 100% NH_3_ conversion efficiency at 330 °C, with N_2_ selectivity exceeding 90% [20]. Kim et al. found that introducing 2 wt.% V into a Pt_0.1_/TiO_2_ (0.1 wt.% Pt) catalyst enables complete NH_3_ conversion over a wide temperature range of 250–350 °C, while achieving a N_2_ selectivity of more than 80% [12].

In our previously published work, Pt-V bimetallic catalysts were prepared by mechanically mixing a VW/TiO_2_ catalyst with a minor quantity of Pt/Al_2_O_3_ catalyst [22]. This catalyst achieved a 100% NH_3_ conversion efficiency at 300 °C, with a N_2_ selectivity of 80.2%. Furthermore, authors investigated the effect of the loading sequence of V and Pt on NH_3_-SCO performance of Pt-V bimetallic catalyst, and found that the V/Pt/Ti catalyst exhibited excellent NH_3_ conversion performance and N_2_ selectivity at low temperatures, enabling complete NH_3_ conversion and a N_2_ selectivity of over 80% at 250 °C [23]. On this basis, authors regulated the synergistic effect between SCO and SCR reactions by adjusting V loading amount, thereby further improving the N_2_ selectivity of the V/Pt/Ti catalyst. The obtained V_0.5_/Pt/TiO_2_ catalyst achieved a N_2_ selectivity of ~90% in a temperature range of 250–450 °C [24]. However, for the adaptation to exhaust temperature (~200 °C) of NH_3_-fueled engines under low-load operating state, the low-temperature activity of the V_0.5_/Pt/TiO_2_ catalyst still needs to be further improved. Previous studies have shown that the activity of a catalyst is strongly correlated with the dispersion and interaction of active components, which are largely dictated by the catalyst preparation method [19,20,21,22,23]. It is highly promising to further improve the low-temperature activity by investigating the influence of preparation approaches on the SCO performance of V-Pt-Ti catalysts, but to our knowledge, no similar literature has been reported so far.

Herein, impregnation (IP), precipitation (PC), sol-gel (SG), thermal decomposition (TD), and hydrothermal (HD) methods (Tables S1–S5) were adopted to prepare V-Pt-Ti catalysts. The influence of preparation methods on the NH_3_-SCO performance of V-Pt-Ti catalysts was systematically investigated. It was revealed by the results that the V-Pt-Ti catalyst prepared by the precipitation method (denoted as VPT-PC) exhibited excellent comprehensive SCO performance. Within the temperature range of 200–450 °C, the VPT-PC catalyst completely converted 3000 ppm NH_3_, and the N_2_ selectivity was higher than 90%. Compared with NH_3_-SCO catalysts reported previously (Table S6), the VPT-PC catalyst demonstrated remarkably superior performance. Some characterization techniques, encompassing N_2_ adsorption/desorption, X-ray diffraction (XRD), X-ray photoelectron spectroscopy (XPS), O_2_ temperature-programmed desorption (O_2_-TPD), H_2_ temperature-programmed reduction (H_2_-TPR), NH_3_ temperature-programmed desorption (NH_3_-TPD), in situ diffuse reflectance infrared Fourier transform spectroscopy (in situ DRIFTS), scanning electron microscopy (SEM), transmission electron microscopy (TEM), were employed to characterize prepared catalysts. Based on the results of characterization, the mechanism by which preparation methods affect catalytic performance were discussed in depth.

## 2. Experimental

### 2.1. Catalyst Preparation

V-Pt-Ti catalysts were prepared via impregnation (IP), precipitation (PC), thermal decomposition (TD), hydrothermal (HD), and sol-gel (SG) methods, and the respective catalysts were denoted as VPT-IP, VPT-PC, VPT-SG, VPT-TD, and VPT-HD. TiO(SO_4_)·xH_2_SO_4_·xH_2_O (AR, 20 wt.% Ti, Aladdin Reagent Co.Ltd., Shanghai, China) was applied as the Ti precursor, while NH_4_VO_3_ and Pt(NO_3_)_2_ (AR, 18.02 wt. % Pt, Aladdin Reagent Co., Ltd., Shanghai, China) were utilized as precursors of V and Pt, respectively. NH_4_OH (AR, 25–28%, Sinopharm Chemical Reagent Co., Ltd., Shanghai, China) and citric acid (C_6_H_8_O_7_, AR, 99.5%, Sinopharm Chemical Reagent Co., Ltd., Shanghai, China) were utilized as the precipitant and complexing agent, respectively. Deionized water (15 MΩ·cm) was employed as the solvent. According to our previous work, the molar ratio of V:Pt:Ti in prepared catalysts was fixed at 0.5:0.01:1 [23,24]. The detailed catalyst preparation procedure and calcination temperature profile (Figure S1) are provided in Supplementary Materials.

### 2.2. Catalyst Characterization

A series of characterization techniques encompassing N_2_ adsorption/desorption, XRD, O_2_-TPD, NH_3_-TPD, SEM, TEM, XPS, and H_2_-TPR were utilized to analyze synthesized catalysts, aimed at elucidating their physicochemical characteristics. The BJH (Barrett–Joyner–Halenda) method was employed to analyze the pore size of catalysts in this work. Furthermore, in situ DRIFTS (Tables S8–S10) was applied to explore reaction intermediates. The details of catalyst characterization are available in Supplementary Materials.

### 2.3. Catalytic Performance Test

The catalytic performance of synthesized catalysts was tested in a fixed-bed reactor, with a catalyst loading volume of 0.2 mL (~0.1 g) for each test. Before the test, a N_2_ flow was applied to flush the catalyst at 100 °C for 30 min to eliminate residual impurities. Next, the reactant gas stream (200 mL/min) was introduced to the reactor, containing 3000 ppm NH_3_ and 5 vol.% O_2_, with N_2_ employed as the balance gas. Thereafter, the reaction temperature was gradually ramped up from 100 to 450 °C with a heating rate of 1.5 °C/min. The concentrations of NH_3_, NO, NO_3_, and N_2_O were measured by an FT-IR spectrometer (IGS, ThermoFisher Scientific, Waltham, MA, USA). Equations (1) and (2) were applied to compute the value of NH_3_ conversion efficiency and N_2_ selectivity.


(1) $${\mathrm{NH}}_{3}\;\mathrm{conversion}=\frac{{\mathrm{NH}}_{3,\mathrm{in}}-{\mathrm{NH}}_{3,\mathrm{out}}}{{\mathrm{NH}}_{3,\mathrm{in}}}\times 100\%$$

(2) $$N_{2}\;\mathrm{selectivity}=\;\frac{{\mathrm{NH}}_{3,\mathrm{in}}-{\mathrm{NH}}_{3,\mathrm{out}}-{\mathrm{NO}}_{\mathrm{out}}-{\mathrm{NO}}_{2,\mathrm{out}}-{2\times N}_{2}O_{\mathrm{out}}}{{\mathrm{NH}}_{3,\mathrm{in}}-{\mathrm{NH}}_{3,\mathrm{out}}}\times 100\%$$

## 3. Results

### 3.1. NH_3_-SCO Performance

**Figure 1:**
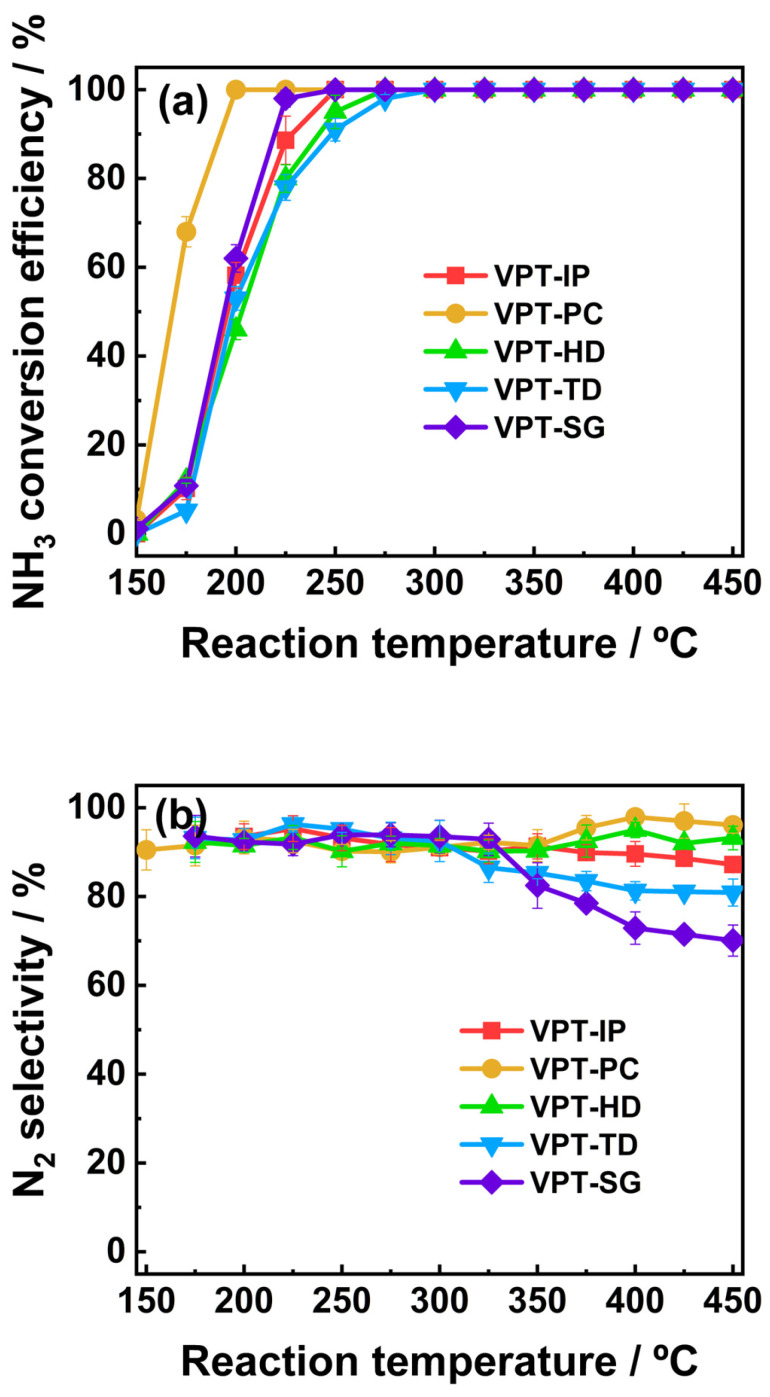
(**a**) NH_3_ conversion efficiency of V-Pt-Ti catalysts; (**b**) N_2_ selectivity of V-Pt-Ti catalysts. Reaction conditions: catalyst volume=0.2 mL (~0.1 g), [NH_3_]in = 3000 ppm, [O_2_]in = 5 vol.%, GHSV = 60,000 h^−1^, and N_2_ was the balance gas.

The NH_3_ conversion performance of the synthesized catalyst across a temperature range of 150–450 °C is presented in Figure 1a. It could be observed that the VPT-PC catalyst achieved a 100% NH_3_ conversion efficiency over a relatively wide temperature range of 200–450 °C. Compared with SCO catalysts reported in prior studies (Table S6), the VPT-PC catalyst exhibited outstanding performance. In contrast, the other catalysts exhibited narrower temperature ranges for complete NH_3_ conversion. The VPT-HD catalyst achieved a 100% NH_3_ conversion within 275–450 °C, the VPT-IP catalyst achieved a 100% NH_3_ conversion within 250–450 °C, the VPT-SG catalyst achieved a 100% NH_3_ conversion within 225–450 °C, and the VPT-TD catalyst achieved a 100% NH_3_ conversion within 300–450 °C. The low-temperature activity of the catalysts prepared via different methods followed the order: VPT-PC > VPT-SG > VPT-IP > VPT-HD > VPT-TD. As shown in Figure 1b, all tested catalysts exhibited N_2_ selectivity above 90% within the temperature range of 150–300 °C. In the range of 300–450 °C, the N_2_ selectivity of VPT-PC and VPT-IP catalysts remained above 90%, while that of VPT-HD, VPT-SG, and VPT-TD catalysts gradually decreased with increasing temperature.

**Figure 2:**
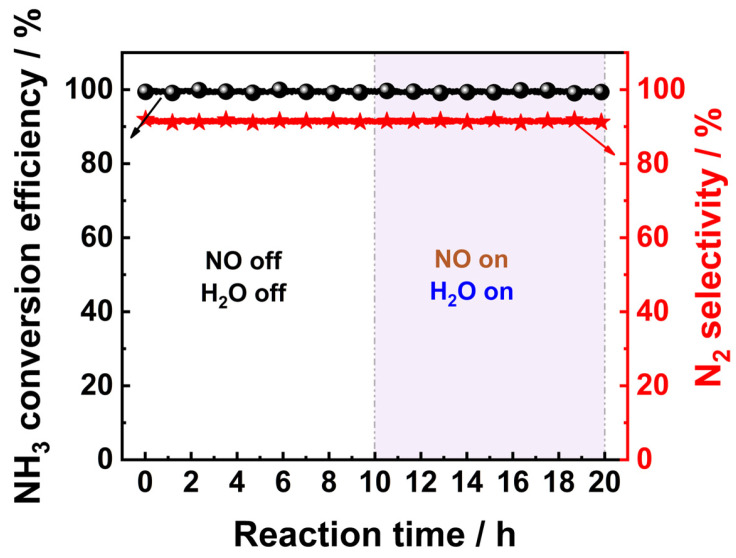
Results of the stability test of the VPT-PC catalyst. Reaction conditions: [NH_3_]in = 3000 ppm, [O_2_]in = 5 vol.%, [NO]in = 500 ppm, [H_2_O]in = 10 vol.%, GHSV = 60,000 h^−1^, T = 200 °C, and N_2_ was the balance gas.

NO and H_2_O are inherent components of real exhaust streams. Thus, the stability of the VPT-PC catalyst was also tested in the presence of 10 vol.% H_2_O vapor and 500 ppm NO at a reaction temperature of 200 °C in 10 h. The results are presented in Figure 2.

It could be seen that in the absence of H_2_O vapor and NO, the NH_3_ conversion and N_2_ selectivity of the VPT-PC catalyst remained stable during long-term continuous operation. The introduction of H_2_O vapor and NO had no significant impact on the NH_3_ conversion and N_2_ selectivity of the VPT-PC catalyst, indicating that the catalyst possessed good stability.

To obtain an in-depth understanding of the mechanism by which different preparation methods influence SCO performance of V-Pt-Ti catalysts, several characterization techniques were employed to characterize physico-chemical properties and surface reaction intermediates of the prepared catalysts. The characterization results were discussed in detail in the following text.

### 3.2. N_2_ Adsorption and Desorption

**Figure 3:**
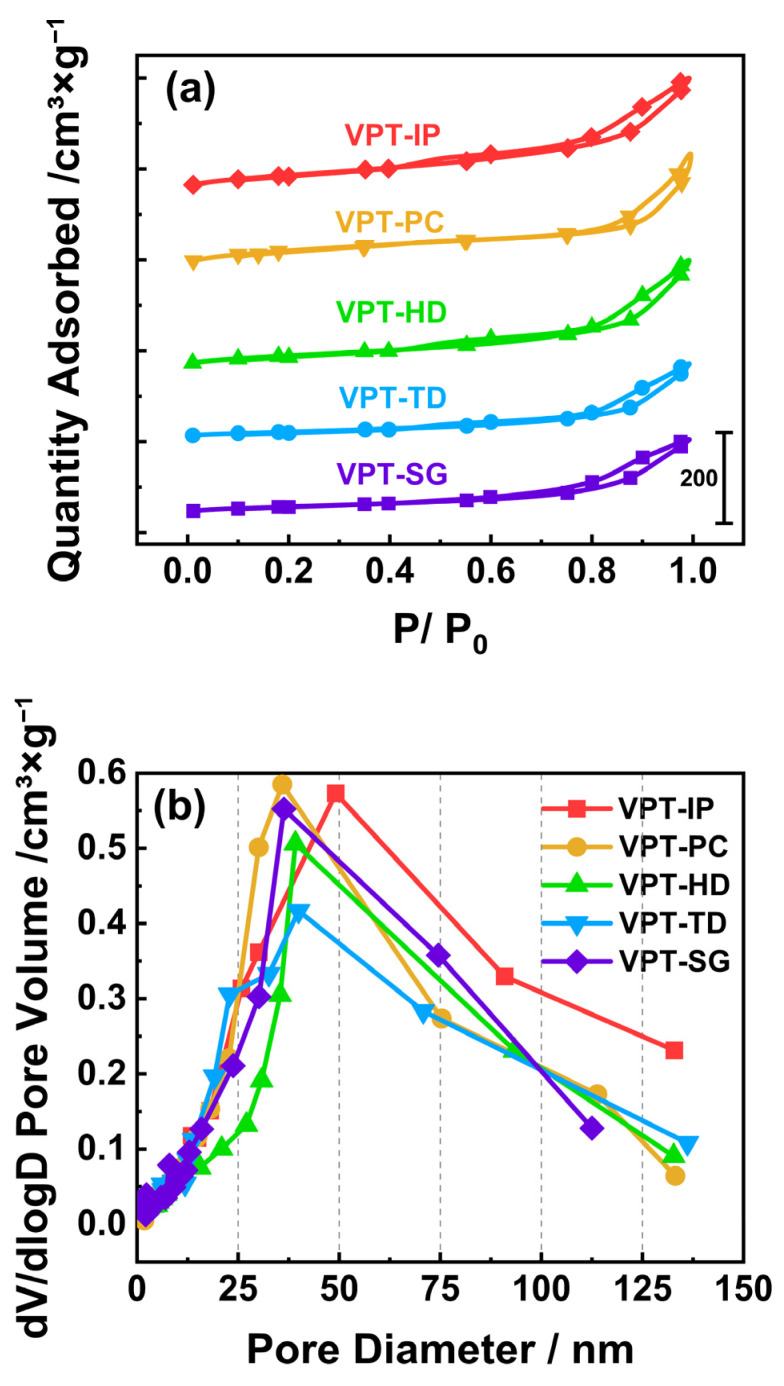
(**a**) N_2_ adsorption–desorption isotherms of V-Pt-Ti catalysts; (**b**) interparticle void size distribution curves of V-Pt-Ti catalysts.

The surface physical properties of catalysts are closely associated with the reactant adsorption and dispersion of active species, thus significantly affecting catalytic performance [25,26]. To characterize the surface physical properties of V-Pt-Ti catalysts, N_2_ adsorption–desorption tests were carried out. N_2_ adsorption–desorption isotherms of the catalysts are shown in Figure 3a. Based on previous reports, the isotherms of tested catalysts can be classified as type IV(a) with H_3_ hysteresis loops [22,23,24]. As can be seen from Figure 3b, the surface interparticle void size of V-Pt-Ti catalysts is mainly around 30–50 nm.

The specific surface areas, interparticle void volumes, and average interparticle void sizes calculated from the adsorption–desorption isotherms are presented in Table 1. It could be seen that the specific surface areas of the tested catalysts exhibited obvious differences, and this was also the case for their interparticle void volumes and average interparticle void sizes. This suggested that the preparation method had a significant impact on physical properties of V-Pt-Ti catalysts. The specific surface areas of the tested catalysts followed the order of VPT-PC > VPT-SG > VPT-IP > VPT-HD > VPT-TD, which was consistent with the order of their low-temperature activity. This suggested that the specific surface area of V-Pt-Ti catalysts was a key factor affecting the low-temperature activity. Generally, a larger specific surface area enables catalysts to adsorb more NH_3_ and O_2_, which is beneficial for enhancing NH_3_-SCO performance [27,28,29]. A large specific surface area is also conducive to achieving a more uniform dispersion of active components on catalyst surface, thereby enriching active sites and improving NH_3_-SCO performance [30]. It could also be observed from Table 1 that the catalysts with higher specific surface areas had relatively smaller interparticle void volumes and average interparticle void sizes, which implied that their higher specific surface areas might be attributed to the presence of more small-sized voids on their surfaces, and this was also consistent with the results in Figure 3b.

### 3.3. XRD

**Figure 4:**
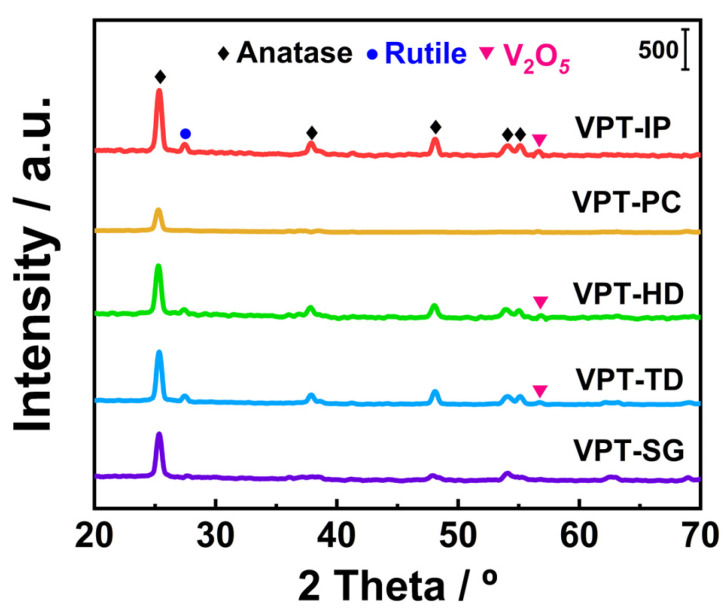
XRD patterns of V-Pt-Ti catalysts.

Figure 4 shows the XRD patterns of the synthesized catalysts. The diffraction peaks centered at 25.4°, 37.9°, 48.1°, 53.91°, 55.0°, and 55.2° could be attributed to the characteristic peaks of anatase TiO_2_ (ICDD 21-1276), while those centered at 27.5° could be attributed to the characteristic peaks of rutile TiO_2_ (ICDD 21-1272) [22,23,24]. The diffraction peaks centered at 56.7° could be ascribed to V_2_O_5_ species (ICDD 19-1401) [30]. As shown in Figure 4, there were a relatively large number of characteristic peaks of TiO_2_ and V_2_O_5_ species with high intensity in XRD patterns of VPT-IP, VPT-TD, and VPT-HD catalysts, but there were only a small number of weak diffraction peaks of anatase in XRD patterns of VPT-PC and VPT-SG catalysts. This indicated that TiO_2_ and V_2_O_5_ species on the surface of VPT-IP, VPT-TD, and VPT-HD catalysts had high crystallinity and large grain sizes, while active components on the surfaces of VPT-PC and VPT-SG catalysts might be in a highly dispersed state or amorphous state.

Quantitative phase analysis was performed on all catalyst samples to further clarify the crystalline phase status of the catalysts. Details of the analysis procedure are provided in Supplementary Materials, and the relevant data are given in Table S7. It could be seen that the crystalline phases on the surfaces of both VPT-PC and VPT-SG catalysts were TiO_2_, and their corresponding signal intensities were significantly lower than those of other Pt-V-Ti catalysts. This implied that VPT-PC and VPT-SG catalysts were largely amorphous.

### 3.4. SEM and TEM

**Figure 5:**
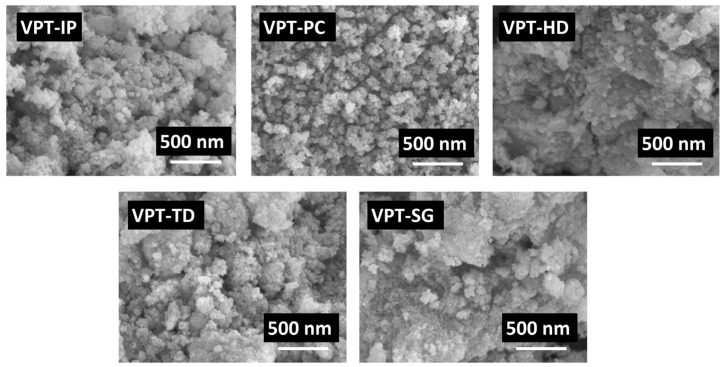
SEM images of V-Pt-Ti catalysts.

The surface morphology of V-Pt-Ti catalysts was characterized by SEM. The SEM images of catalyst samples are shown in Figure 5. It could be observed that the particle sizes of VPT-PC and VPT-SG catalysts were smaller than those of VPT-IP, VPT-TD, and VPT-HD catalysts. This indicated that V-Pt-Ti catalysts synthesized via the precipitation (PC) and sol-gel (SG) methods might exhibit better dispersibility, while those synthesized by impregnation (IP), thermal decomposition (TD), and hydrothermal (HD) methods might have relatively higher crystallinity.

TEM and EDS characterizations were carried out to analyze the distribution of O, Pt, V, and Ti elements on the surface of the VPT-PC catalyst. The results are displayed in Figure S2. It could be seen that O, Pt, V, and Ti elements were relatively evenly dispersed on the surface of the VPT-PC catalyst. Pt and V elements maintained close contact, which effectively promoted the synergistic interaction between Pt and V species.

### 3.5. NH_3_-TPD

**Figure 6:**
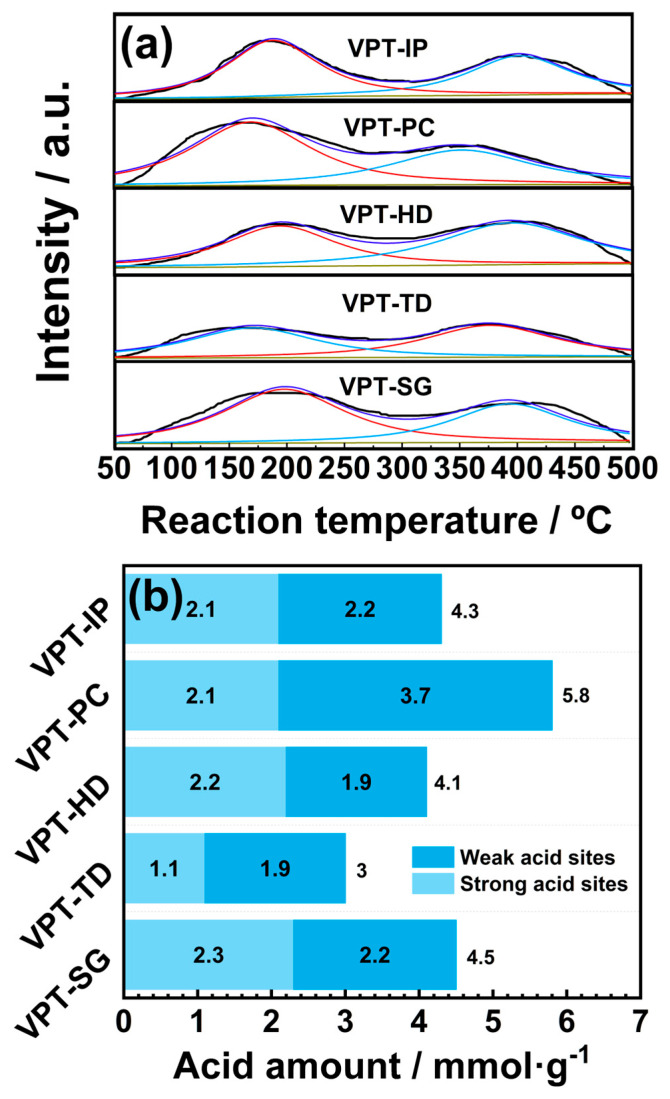
(**a**) NH_3_-TPD curves of V-Pt-Ti catalysts; (**b**) acid site amounts of V-Pt-Ti catalysts.

Surface acid sites perform a vital role in adsorption and activation of NH_3_ [31]. NH_3_-TPD experiments are often conducted to characterize the surface acid sites of synthesized catalysts. The NH_3_-TPD experimental results of V-Pt-Ti catalysts are presented in Figure 6. As presented in Figure 6a, the NH_3_-TPD curves of all catalysts exhibited a similar shape, with two distinct NH_3_ desorption peaks. The peak centered at low temperatures (below 300 °C) was attributed to the signal of NH_3_ species desorbed from weak acid sites, while the peak dominated at high temperatures (above 300 °C) was attributed to the signal of NH_3_ species desorbed from strong acid sites [22]. The amount of acid sites on the surface of each catalyst could be calculated by integrating sub-peak areas in NH_3_-TPD curves [31,32,33], and the results are shown in Figure 6b. It could be noted that the total acid amounts of V-Pt-Ti catalysts prepared by different methods varied significantly, following the order of VPT-PC > VPT-SG > VPT-IP > VPT-HD > VPT-TD, which matched the order of their low-temperature activity from highest to lowest. This indicated that different preparation methods exerted a significant influence on the surface acid amounts of V-Pt-Ti catalysts, thereby affecting their low-temperature catalytic activity.

### 3.6. XPS

**Figure 7:**
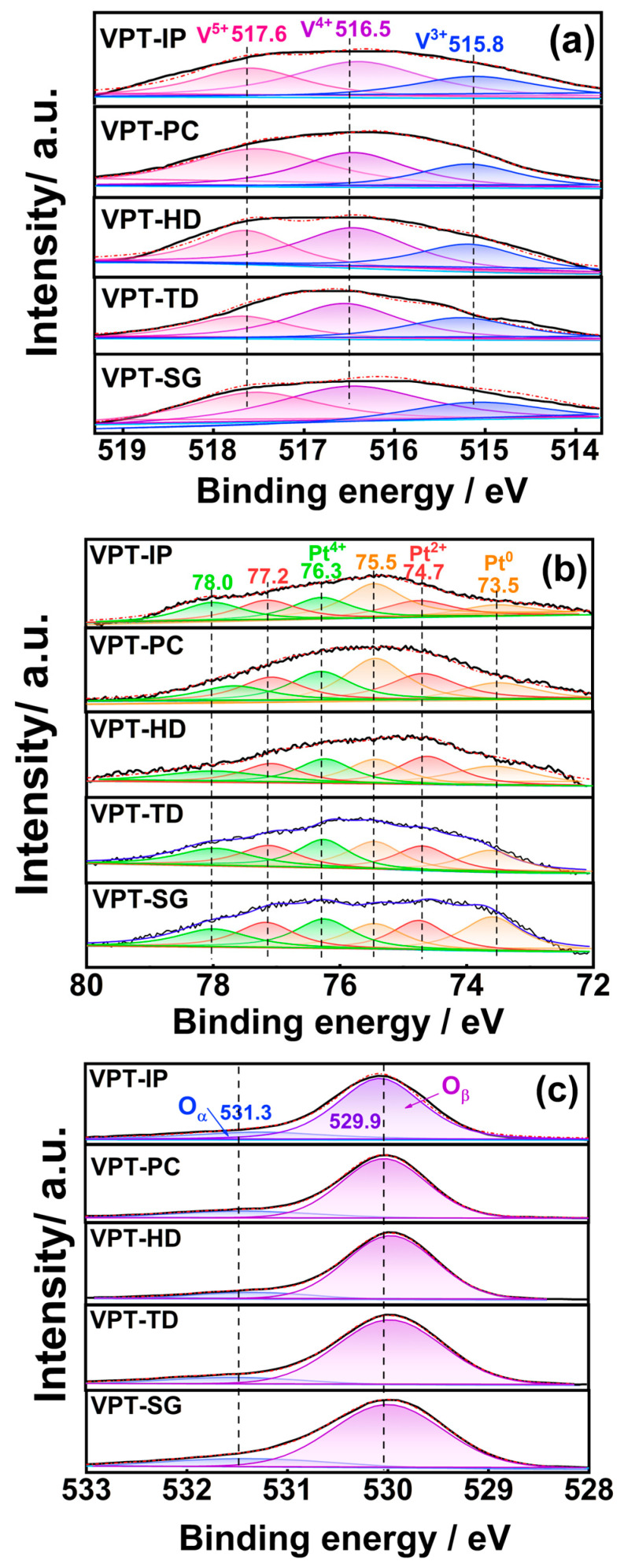
V 2p (**a**), Pt 4f (**b**), and O 1s (**c**) XPS spectra of V-Pt-Ti catalysts.

To investigate the valence states and relative content of main elements on the surface of the prepared catalysts, XPS measurements were carried out, and the tested elements were Pt, V and O. The XPS spectra of tested catalysts are shown in Figure 7. The relative contents of surface elements, which were calculated by integrating corresponding sub-peaks in the spectra, are given in Table S11. It could be seen from Table S11 and Figure 7 that the XPS test results of V-Pt-Ti catalysts prepared by different methods varied significantly from one another, which indicated that the preparation methods had a great influence on the surface chemical properties of the catalysts.

As illustrated in Figure 7a, the V 2p spectra of each V-Pt-Ti catalyst could be divided into three sub-peaks. The sub-peaks centered at 515.8, 516.5, and 517.6 eV were attributed to V^3+^, V^4+^, and V^5+^ species, respectively [23,24,25]. According to Table S11, the order of V^5+^/(V^3+^, V^4+^, and V^5+^) ratios for V-Pt-Ti catalysts prepared by different methods was VPT-PC > VPT-SG > VPT-IP > VPT-HD > VPT-TD. It was reported that V^5+^ species possess excellent NH_3_-SCR catalytic activity [30]. The relatively high V^5+^/(V^3+^+V^4+^+V^5+^) ratio of VPT-PC catalyst was beneficial for rapidly converting the NO_x_ (generated by catalytic oxidation of NH_3_ by Pt species) into N_2_, thereby enabling the VPT-PC catalyst to demonstrate excellent SCO performance.

The Pt 4f spectra of V-Pt-Ti catalysts are shown in Figure 7b. It could be observed that each spectrum could be decomposed into four sub-peaks. The sub-peaks located from 73.5 to 78.0 eV could be attributed to Pt^0^, Pt^2+^, and Pt^4+^ species, as marked in Figure 7b [23,30]. As shown in Table S11, the order of Pt^0^/(Pt^0^+Pt^2+^+Pt^4+^) ratios on the surface of tested catalysts was VPT-PC > VPT-SG > VPT-IP > VPT-HD > VPT-TD. In comparison to Pt^2+^ and Pt^4+^ species, Pt^0^ had superior catalytic oxidation activity, mainly due to its high effectiveness in O_2_ dissociation [24,25]. The presence of a relatively large amount of Pt^0^ species was conducive to facilitating the oxidation of NH_3_ to NO_x_ by the VPT-PC catalyst, which in turn enhanced NH_3_-SCO performance via i-SCR mechanism.

Figure 7c shows the O 1s spectra of all V-Pt-Ti catalysts. It was noteworthy that each spectrum in the figure could be split into two sub-peaks. The sub-peak at 531.3 eV corresponded to chemically adsorbed oxygen species (O_α_), while that at 529.9 eV was ascribed to lattice oxygen species (O_β_). As presented in Table S11, the VPT-PC catalyst had an O_α_/(O_α_+O_β_) ratio of 15.26%, which was higher than those of other V-Pt-Ti catalysts. This was consistent with O_2_-TPD results. In SCO reaction, O_α_ species generally show higher activity than O_β_ species, which can be attributed to their high mobility [31,32,33]. Thus, the relatively higher abundance of O_α_ species might be a key factor contributing to the outstanding SCO performance of the VPT-PC catalyst.

### 3.7. H_2_-TPR

**Figure 8:**
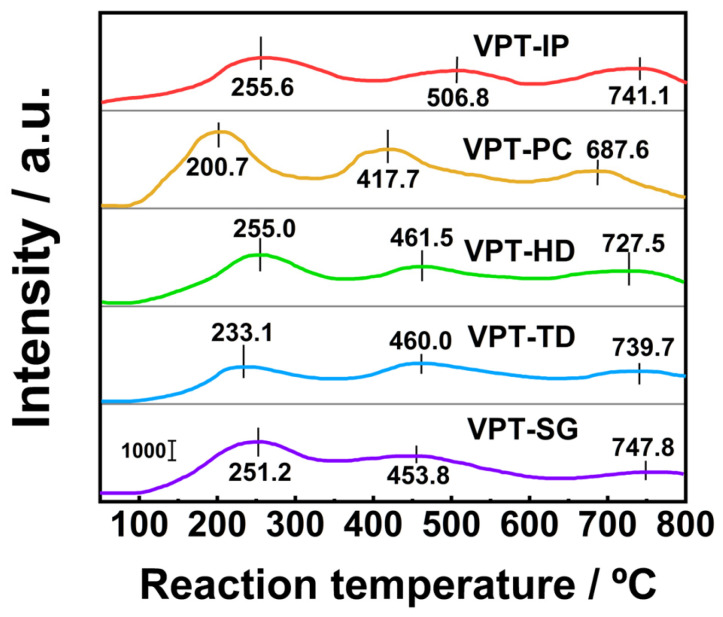
H_2_-TPR profiles of V-Pt-Ti catalysts.

The NH_3_-SCO activity of catalysts was strongly associated with their redox property. To explore the redox property of the prepared catalysts, H_2_-TPR experiments were performed across the temperature range of 50 to 800 °C, and the results are shown in Figure 8. It could be seen that there were obvious peaks in the H_2_-TPR profiles of V-Pt-Ti catalysts prepared by different methods, but the shapes of profiles varied significantly, indicating that the preparation method had a significant influence on the redox property. The reduction peaks at 255.6, 200.7, 255.0, 233.1, and 251.2 °C were attributed to the reduction processes of Pt^2+^ species to Pt^0^ species and V^5+^ species to V^4+^ species [20,30]. The reduction of V^4+^ species to V^3+^ species and subsequent reduction of V^3+^ species resulted in reduction peaks at 506.8, 417.7, 461.5, 460.0, and 453.8 °C [30,34]. Since TiO_2_ hardly reacted with H_2_ at 600 °C, the reduction peaks at 741.1, 687.6, 727.5, 739.7, and 747.8 °C were ascribed to the reduction of TiO_2_ species [34,35]. It could be seen from Figure 8 that the temperatures corresponding to reduction peaks in the H_2_-TPR curve of VPT-PC catalyst were lower than those of other catalysts. This indicated that the VPT-PC catalyst possessed better redox property compared with other V-Pt-Ti catalysts. It could be seen from Table S12 that the H_2_ consumption value of the VPT-PC catalyst (0.79 mmol/g) was higher than those of other V-Pt-Ti catalysts. This indicated that there were more abundant reducible species on the surface of the VPT-PC catalyst compared with those of other V-Pt-Ti catalysts. According to previous studies [36,37,38], the excellent redox properties and abundant reducible species were beneficial to the VPT-PC catalyst exhibiting excellent NH_3_-SCO activity.

### 3.8. O_2_-TPD

**Figure 9:**
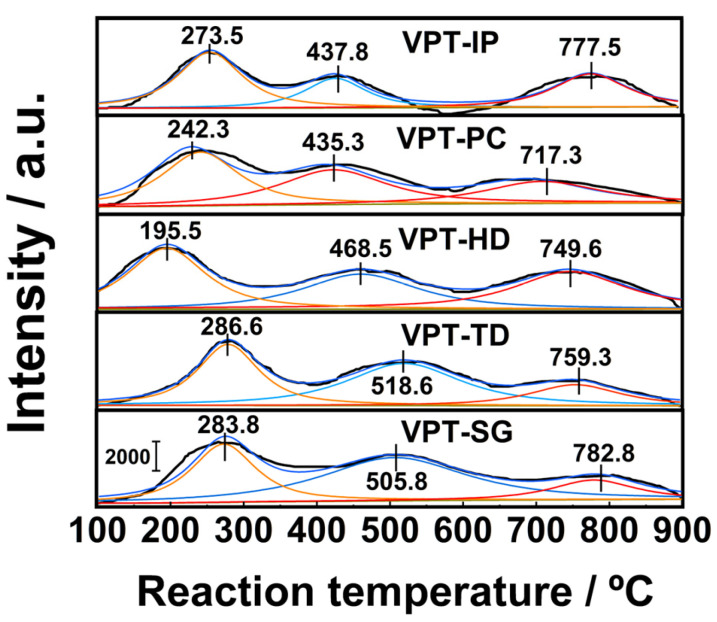
O_2_-TPD profiles of V-Pt-Ti catalysts.

The surface oxygen species of catalysts are capable of participating in catalytic oxidation reactions of NH_3_, in turn influencing NH_3_-SCO performance of catalysts. With the aim of characterizing oxygen species on the surfaces of V-Pt-Ti catalysts, O_2_-TPD tests were carried out, and the results are presented in Figure 9. It could be seen that each catalyst displayed three well-defined oxygen desorption peaks in its O_2_-TPD profile. It was reported that oxygen desorption peaks below 250 °C were primarily due to the desorption of oxygen species with physical adsorption [38,39,40]. The oxygen desorption peaks in the range of 250–600 °C were mainly caused by the desorption of chemically adsorbed surface oxygen from oxygen vacancies. In addition, oxygen desorption between 600 and 900 °C was associated with the desorption of surface lattice oxygen.

As shown in Figure 9, the temperatures corresponding to the peaks of chemically adsorbed surface oxygen and surface lattice oxygen species in the O_2_-TPD curve of the VPT-PC catalyst were 435.3 °C and 717.3 °C, respectively, which were significantly lower than those of the other V-Pt-Ti catalysts in the same temperature range. This indicated that, compared with other catalysts, the oxygen vacancies and lattice oxygen species on the surface of the VPT-PC catalyst might possess higher activity [41,42,43]. This was beneficial for facilitating adsorption and activation of O_2_ and promoting the migration of active oxygen on the catalyst surface, thereby enhancing the NH_3_-SCO activity of the catalyst.

### 3.9. In Situ DRIFTS

Numerous literature reports indicated that the adsorption and activation of NH_3_ on the catalyst surface are prerequisites for NH_3_-SCO reactions [42,43,44,45]. Thus, in situ DRIFTS experiments involving the pre-adsorption of NH_3_ followed by the subsequent adsorption of O_2_ were conducted. The steps of in situ DRIFTS experiments were as follows: Initially, the catalysts were purged with N_2_ at a flow rate of 100 mL/min at 200 °C. Subsequently, the catalysts were subjected to the pre-adsorption of NH_3_ with a concentration of 3000 ppm at 100 mL/min for 30 min to achieve saturation. After that, they were rinsed with N_2_ (100 mL/min) for 30 min to remove physically adsorbed NH_3_. Finally, 5% O_2_ (100 mL/min) was fed into the reactor, and IR spectra were recorded over time.

**Figure 10:**
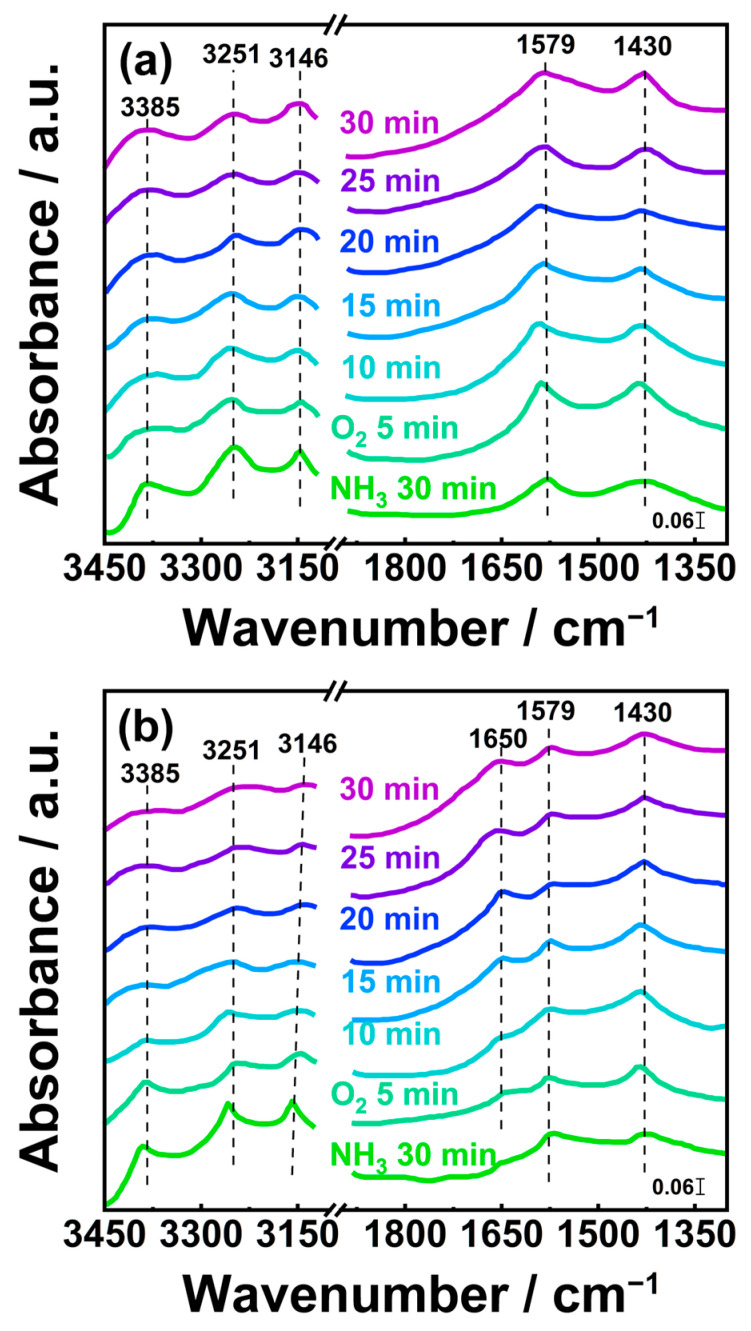

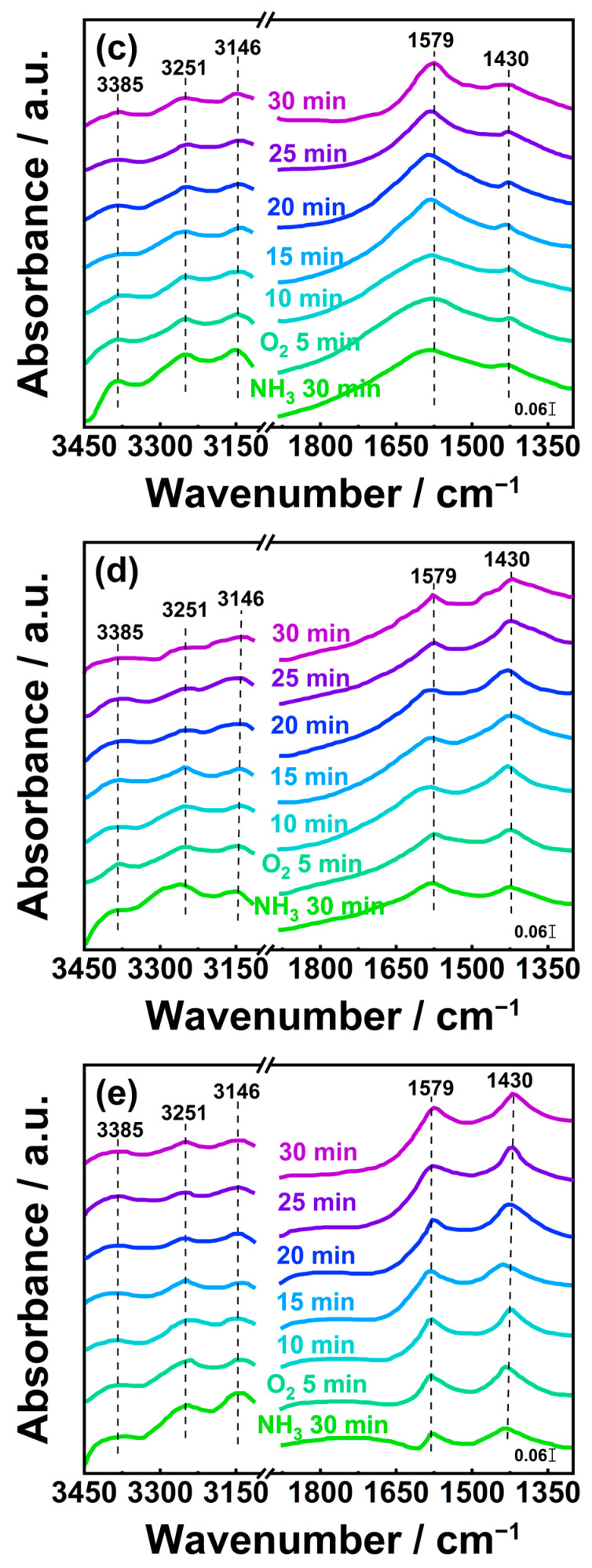
In situ DRIFT spectra of VPT-IP (**a**), VPT-PC (**b**), VPT-HD (**c**), VPT-TD (**d**), and VPT-SG (**e**) catalysts.

Figure 10 displays in situ DRIFT spectra obtained during the reaction of pre-adsorbed NH_3_ species with introduced O_2_ on the surface of V-Pt-Ti catalysts. As shown in Figure 10, after O_2_ was added to the feed gas, the intensity of bands that could be assigned to coordinated NH_3_ on Lewis acid sites (3385, 3251 cm^−1^), and N-H stretching vibration modes of coordinated NH_3_ species (3146 cm^−1^) decreased gradually. The intensity of bands corresponding to bidentate nitrate species (1579 cm^−1^) and NH_4_^+^ species coordinated at Brønsted acid sites (1430 cm^−1^) increased gradually [46,47,48]. This indicated that the NH_3_ coordinated on Lewis acid sites and NH_3_ species coordinated on the surface of V-Pt-Ti catalysts were able to react with O_2_, generating NH_4_^+^ species coordinated at Brønsted acid sites (1436 cm^−1^) species and bidentate nitrate species (1579 cm^−1^) [23,24,25].

Notably, as depicted in Figure 10, when O_2_ was introduced into the reaction cell, a clear peak corresponding to NO_2_ species (1650 cm^−1^) emerged in the in situ DRIFTS spectrum of the VPT-PC catalyst [49]. In contrast, this peak was absent in the in situ DRIFTS spectra of other V-Pt-Ti catalysts under the same reaction conditions. This observation suggested that NO_2_ species might be involved in the NH_3_-SCO reaction over the VPT-PC catalyst. Since that NO_2_ species acted as a key reactant in i-SCR reactions, as shown in Equations (3) and (4) [50,51], their presence on the surface of the VPT-PC catalyst might have contributed to the promotion of i-SCR reactions, which facilitated the catalyst in exhibiting excellent SCO performance at 200 °C.


(3) $${\mathrm{NH}}_{3}+O*(\mathrm{reactive}\;\mathrm{oxygen}\;\mathrm{species})\to {-\mathrm{NH}}_{2}+-\mathrm{OH}$$

(4) $${-\mathrm{NH}}_{2}+{\mathrm{NO}}_{2}+O*(\mathrm{reactive}\;\mathrm{oxygen}\;\mathrm{species})\to 2N_{2}+3H_{2}O$$

As shown in Figure S3, when O_2_ was introduced into the reaction cell, a clear peak corresponding to NO_2_ species (1650 cm^−1^) emerged in the in situ DRIFTS spectrum of PT catalyst, which is consistent with the case of the VPT-PC catalyst. However, this peak was absent in the in situ DRIFTS spectra of other V-Pt-Ti catalysts under the same reaction conditions. This suggested that the presence of NO_2_ species on the surface of the VPT-PC catalyst might be related to Pt species.

## 4. Discussion

In summary, different preparation methods could exert a comprehensive influence on the surface texture, crystal structure, acid sites, surface element distribution, redox properties, and surface reaction pathways of V-Pt-Ti catalysts, which in turn significantly affected the SCO performance of the catalysts.

N_2_ adsorption and desorption test results showed that various preparation methods had a significant impact on the specific surface area and surface interparticle void size of V-Pt-Ti catalysts. In comparison with other V-Pt-Ti catalysts, the VPT-PC catalyst possessed a higher specific surface area. This not only facilitated the VPT-PC catalyst in efficiently adsorbing reactants but also promoted the formation of more abundant active sites, thus enabling the VPT-PC catalyst to demonstrate outstanding SCO performance.

The XRD test results showed that different preparation methods significantly affected the crystallinity of V-Pt-Ti catalysts. In comparison with other V-Pt-Ti catalysts, the VPT-PC catalyst had lower crystallinity; this favored the creation of a greater number of surface lattice defects [50], thereby producing more abundant and highly active oxygen vacancies as well as reducible species. This was beneficial for the VPT-PC catalyst to exhibit excellent redox properties, which matched the findings from H_2_-TPR and O_2_-TPD tests. 

The results of NH_3_-TPD tests indicated that different preparation methods could affect SCO performance by influencing the total surface acid amounts of V-Pt-Ti catalysts. Compared with other V-Pt-Ti catalysts, the VPT-PC catalyst had more abundant surface acid sites, thus exhibiting better SCO performance. 

The XPS test results showed that different preparation methods could affect the catalyst performance by influencing the relative contents of Pt^0^, V^5+^, and O_α_ on the surface of V-Pt-Ti catalysts. In comparison with other V-Pt-Ti catalysts, the VPT-PC catalyst had higher relative contents of Pt^0^, V^5+^, and O_α_, thereby demonstrating better SCO performance.

The results of in situ DRIFTS tests indicated that NO_2_ species could participate in the NH_3_ oxidation reaction on the surface of the VPT-PC catalyst, whereas this was not the case for other V-Pt-Ti catalysts. As shown in Equation (3), NO_2_ species are key reactants in i-SCR reactions. Thus, the presence of NO_2_ species was able to facilitate the VPT-PC catalyst in exhibiting excellent SCO performance at 200 °C by promoting i-SCR reactions.

To determine whether i-SCR reaction dominated SCO reaction over the VPT-PC catalyst, a stability test on the PT catalyst (Table S8) was conducted for comparison. The concentrations of NH_3_, NO, NO_2_, and N_2_O at the outlet of the reactor during the test are shown in Figures S4 and S5. It could be seen from that the PT catalyst could achieve a complete conversion of NH_3_ at 200 °C, while the concentration of NO_2_ generated during the reaction is much higher than those of NO and N_2_O.

It could be observed from the comparison of Figure S4 and Figure S5 that the introduction of V resulted in a decrease in outlet NO_2_ concentration from ~1150 ppm to 0 ppm, and a decrease in NO concentration from ~300 ppm to ~200 ppm. The decrease in NO_2_ concentration was significantly higher than that of NO.

Previous studies showed that NO_2_ species could participate in SCR reaction under the action of V species, thereby being converted into N_2_ and H_2_O, as shown in Equation (5) [25,26]. This indicated that a large amount of NO_2_ was converted via SCR reaction after the introduction of V species into the PT catalyst. Given that the outlet NO_2_ is mainly derived from NH_3_ oxidation, it was evident that i-SCR reaction dominates NH_3_-SCO reaction over the VPT-PC catalyst.


(5) $$\mathrm{NO}+{\mathrm{NO}}_{2}+2{\mathrm{NH}}_{3}\;\text{→}\;2N_{2}+3H_{2}O$$

## 5. Conclusions

Herein, a series of V-Pt-Ti catalysts were synthesized using impregnation (IP), precipitation (PC), sol-gel (SG), thermal decomposition (TD), and hydrothermal (HD) methods. The results showed that different preparation methods led to notable differences in physico-chemical property of the catalysts. In detail, diverse preparation methods caused significant alterations to surface structural characteristics, crystallinity, chemical states of elements, surface acid site amounts, and redox property of V-Pt-Ti catalysts, thereby exerting a great influence on SCO performance. The catalyst synthesized via the PC method exhibited better low-temperature SCO activity and N_2_ selectivity compared with those prepared by the other four methods. This might be primarily attributed to the improved specific surface area, surface acidity, surface active elements, and redox property of the VPT-PC catalyst. According to the results of in situ DRIFTS, NO_2_ species could participate in NH_3_ oxidation reaction on the surface of the VPT-PC catalyst, which was beneficial for improving SCO activity.

## Figures and Tables

**Table 1:** Specific surface areas and average interparticle void sizes of V-Pt-Ti catalysts.

Sample	S_BET_ (m^2^/g)	Void Volume (cm^2^/g)	Void Diameter (nm)
VPT-IP	61.83	0.36	25.67
VPT-PC	66.22	0.31	20.65
VPT-HD	58.27	0.32	23.78
VPT-TD	51.63	0.33	22.95
VPT-SG	62.88	0.32	23.92

## Data Availability

The original contributions presented in this study are included in the article/Supplementary Materials. Further inquiries can be directed to the corresponding author.

## Supplementary Materials

The following supporting information can be downloaded at: https://www.mdpi.com/article/10.3390/ma19010194/s1, Figure S1: Calcination temperature profile of V-Pt-Ti catalysts; Figure S2: TEM and EDS image of the VPT-PC catalyst; Figure S3: In situ DRIFT spectra of the VT (a), PT (b), and T (c) catalysts; Figure S4: The stability test results of the VPT-PC catalyst. Reaction conditions: [NH_3_]in = 3000 ppm, [O_2_]in = 5 vol.%, GHSV = 60,000 h^−1^, T = 200 °C, and N_2_ was the balance gas; Figure S5: The stability test results of the PT catalyst. Reaction conditions: [NH_3_]in = 3000 ppm, [O_2_]in = 5 vol.%, GHSV = 60,000 h^−1^, T = 200 °C, and N_2_ was the balance gas; Table S1: Synthetic procedures and technological parameters of the VPT-IP catalyst; Table S2: Synthetic procedures and technological parameters of the VPT-PC catalyst; Table S3: Synthetic procedures and technological parameters of the VPT-TD catalyst; Table S4: Synthetic procedures and technological parameters of the VPT-TD catalyst; Table S5: Synthetic procedures and technological parameters of the VPT-TD catalyst; Table S6: NH_3_-SCO performance comparison for the VPT-PC catalyst and other catalysts in previous work; Table S7: Synthetic procedures and technological parameters of the VPT-TD catalyst; Table S8: Synthetic procedures and technological parameters of the PT catalyst; Table S9: Synthetic procedures and technological parameters of the VT catalyst; Table S10: Synthetic procedures and technological parameters of the T catalyst; Table S11: XPS results of V-Pt-Ti catalysts; Table S12: H_2_ consumption values of V-Pt-Ti catalysts. References [12,20,23,29,48,52,53,54,55,56,57,58,59,60,61,62,63,64] have cited in the Supplementary Materials.
