# Supplementary material for: Boosted NH3 Selective Catalytic Oxidation Activity over V-Pt-Ti Catalysts: Insight into Preparation Method Effects
Source: Materials (Basel). 2026 Jan 5;19(1):194. doi: 10.3390/ma19010194 (PMC12786668; doi:10.3390/ma19010194)
Supplement: Supplementary file 1 [file materials-19-00194-s001.zip › materials-4021373-supplementary.pdf]

materials

MDPI

*Supplementary Material*

# Boosted $NH_3$ Selective Catalytic Oxidation Activity over V-Pt-Ti Catalysts: Insight into Preparation Method Effects

**Yu Gao, Lipeng Wang *, Kun Li and Yongbo Ji**

China Waterborne Transport Research Institute, Beijing 100088, China; gaoyu@wti.ac.cn (Y.G.); likun@wti.ac.cn (K.L.); jyb@wti.ac.cn (Y.J.)

* Correspondence: wanglp@wti.ac.cn

## Section S1. Catalysts preparation

V-Pt-Ti catalysts were prepared via impregnation (IP), precipitation (PC), thermal decomposition (TD), and hydrothermal (HD), and sol-gel (SG) methods, and the respective catalysts were denoted as VPT-IP, VPT-PC, VPT-SG, VPT-TD, and VPT-HD. $TiO(SO_4){\cdot}xH_2SO_4{\cdot}xH_2O$ (AR, 20 wt.% Ti, Aladdin Reagent Co.Ltd., Shanghai, China) was applied as the Ti precursor, while $TiO_2$ powder (Degussa P25) was used as the support of the VPT-IP catalyst. $NH_4VO_3$ (AR, 99.8 %, Aladdin Reagent Co.Ltd., Shanghai, China) and $Pt(NO_3)_2$ (AR, 18.02 wt. % Pt, Aladdin Reagent Co.Ltd., Shanghai, China) were utilized as precursors of V and Pt, respectively. $NH_4OH$ (AR, 25–28%, Sinopharm Chemical Reagent Co.Ltd., Shanghai, China) and citric acid ($C_6H_8O_7$, AR, 99.5%, Sinopharm Chemical Reagent Co.Ltd., Shanghai, China) were utilized as the precipitant and complexing agent, respectively. Deionized water (15 MΩ·cm) was employed as the solvent. According to our previous work, the molar ratio of V:Pt:Ti in prepared catalysts was fixed at 0.5:0.01:1 [1].

**Impregnation (IP):**

The VPT-IP catalyst was prepared by impregnating a $Pt/TiO_2$ catalyst in a V precursor solution. The specific process consisted of two stages; the specific steps are shown in Table S1.

**Table S1.** Synthetic procedures and technological parameters of the VPT-IP catalyst.

| Stages | Steps | Contents |
| --- | --- | --- |
| 1. Synthesis of the $Pt/TiO_2$ catalyst | 1-1 | A $Pt(NO_3)_2$ solution (0.27 g) was directly added to 100 mL of deionized water. |
| | 1-2 | The mixture was subjected to continuous stirring (150 rpm) at room temperature for 10 min. |
| | 1-3 | $TiO_2$ powder (2 g) was directly added to the solution obtained from the previous step. |
| | 1-4 | The mixture was subjected to continuous stirring (150 rpm) for 2 h at room temperature. |
| | 1-5 | The mixture was put in a rotary evaporator, and rotary evaporation dehydration was conducted at 80 °C in an air atmosphere at a rotation speed of 100 rpm for 1 h. |

| | | |
|---|---|---|
| | 1-6 | The mixture was put in a blast drying oven, and blast drying was performed on it at 120 °C for 6 h in an air atmosphere. |
| | 1-7 | The resulting solid was calcined for 3 h in an air atmosphere, and the calcination temperature profile is shown in Figure S1. |
| | 2-1 | $NH_4VO_3$ (1.46 g) was directly added to 100 ml deionized water. |
| | 2-2 | The mixture was subjected to continuous stirring (150 rpm) at 60 °C for 30 min. |
| | 2-3 | All of the $Pt/TiO_2$ catalysts synthesized in the previous stage was directly added to the solution obtained from the previous step. |
| | 2-4 | The mixture obtained from the previous step was stirred continuously (200 rpm) for 2 h at room temperature. |
| 2. Synthesis of VPT-IP catalyst | 2-5 | The mixture was put in a rotary evaporator, and dehydration was conducted by rotary evaporation at 80 °C in an air atmosphere at 100 rpm for 1 h. |
| | 2-6 | The mixture obtained after rotary evaporation dehydration was put in a blast drying oven, and blast drying was performed on it at 120 °C for 6 h in an air atmosphere. |
| | 2-7 | The solid obtained after drying was put in a muffle furnace and calcined for 3 h in an air atmosphere, and the calcination temperature profile is shown in Figure S1. |
| | 2-8 | The solid obtained after calcination was ground, tableted and sieved into particles of 40−60 mesh, which can be directly used for activity testing. |

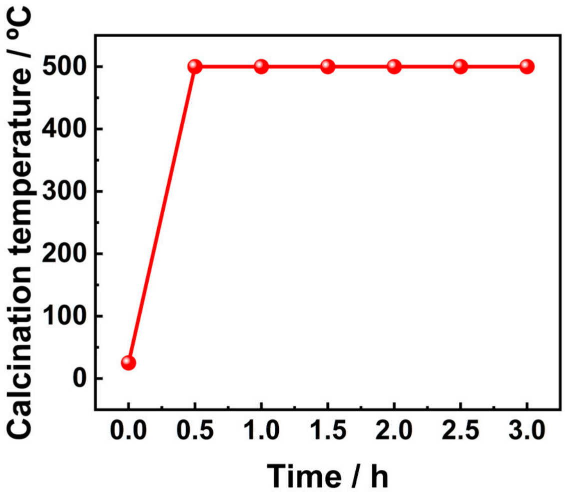


**Figure S1.** Calcination temperature profile of V-Pt-Ti catalysts.

**Precipitation (PC):**

The VPT-PC catalyst was prepared by the precipitation method, and the specific steps are shown in Table S2.

**Table S2.** Synthetic procedures and technological parameters of the VPT-PC catalyst.

| Steps | Contents |
|---|---|
| 1 | A $Pt(NO_3)_2$ solution (0.27 g) and $NH_4VO_3$ (1.46 g) were added simultaneously to 100 mL of deionized water. |
| 2 | The mixture was subjected to continuous stirring (200 rpm) at 60 ºC for 30 min. |
| 3 | $TiO(SO_4)\cdot xH_2SO_4\cdot xH_2O$ (6 g) was added to the mixture. |
| 4 | $NH_4OH$ was added dropwise to the mixture obtained from the previous step with continuous stirring (150 rpm) until the pH value of the mixture reached 10. |
| 5 | The obtained suspension solution was statically placed at room temperature in an air atmosphere for 12 h. |
| 6 | The precipitate was put in a blast drying oven, and blast drying was performed on it at 120 ºC for 6 h in an air atmosphere. |
| 7 | The solid obtained after drying was put in a muffle furnace and calcined for 3 h in an air atmosphere; the calcination profile is shown in Figure S1. |
| 8 | The solid obtained after calcination was ground, tableted and sieved into particles of 40−60 mesh, which can be directly used for activity testing. |

**Thermal decomposition (TD):**

The VPT-TD catalyst was prepared by the thermal decomposition method, and the specific steps are shown in Table S3.

**Table S3.** Synthetic procedures and technological parameters of the VPT-TD catalyst.

| Steps | Contents |
|---|---|
| 1 | A $Pt(NO_3)_2$ solution (0.27 g), $NH_4VO_3$ (1.46 g), and $TiO(SO_4)\cdot xH_2SO_4\cdot xH_2O$ (6 g) were added simultaneously to 100 mL of deionized water. |
| 2 | The mixture was subjected to continuous stirring (200 rpm) at 60 ºC for 1 h. |
| 3 | The resulting solution was put in a blast drying oven, and blast drying was performed on it at 120 ºC for 6 h in an air atmosphere. |
| 4 | The obtained crystallized solid was put in a muffle furnace and calcined for 3 h at 500 ºC in an air atmosphere; the calcination profile is shown in Figure S1. |
| 5 | The solid obtained after calcination was ground, tableted and sieved into particles of 40−60 mesh, which can be directly used for activity testing. |

**Hydrothermal (HD):**

The VPT-HD catalyst was prepared by the thermal decomposition method, and the specific steps are shown in Table S4.

**Table S4.** Synthetic procedures and technological parameters of the VPT-TD catalyst.

| Steps | Contents |
|---|---|
| 1 | A $Pt(NO_3)_2$ solution (0.27 g), $NH_4VO_3$ (1.46 g), and 6 g of $TiO(SO_4)\cdot xH_2SO_4\cdot xH_2O$ were added in 100 mL of deionized water. |
| | The mixture was subjected to continuous stirring (150 rpm) at 60 ºC for 1 h. |
| 2 | The mixture was transferred into 200 mL Teflon-lined stainless steel autoclaves and sealed. |
| 3 | The autoclaves were placed in an oven to maintain the temperature at 150 ºC for 10 h. |
| 4 | After the autoclave was cooled to room temperature, the precipitate was collected, filtered and washed with deionized water until neutral. |
| 5 | The precipitate was put in a blast drying oven, and blast drying was performed on it at 120 ºC for 6 h in an air atmosphere. |
| 6 | The obtained solid was put in a muffle furnace and calcined for 3 h at 500 ºC in an air atmosphere; the calcination profile is shown in Figure S1. |
| 7 | The solid obtained after calcination was ground, tableted and sieved into particles of 40−60 mesh, which can be directly used for activity testing. |

**Sol-gel (SG):**

The VPT-SG catalyst was prepared by the sol-gel method, and the specific steps are shown in Table S5.

**Table S5.** Synthetic procedures and technological parameters of the VPT-TD catalyst.

| Steps | Contents |
|---|---|
| 1 | $Pt(NO_3)_2$ solution (0.27 g), 0.65 g $C_6H_8O_7$, 1.46 g $NH_4VO_3$, and 6 g of $TiO(SO_4)\cdot xH_2SO_4\cdot xH_2O$ were added in 100 mL of deionized water. |
| 2 | The mixture was subjected to continuous stirring (150 rpm) at 60 ºC for 1 h. |
| 3 | $NH_4OH$ was added dropwise into the mixture until the pH value reached 10. |
| 4 | The mixture was subjected to continuous stirring (150 rpm) at 80 ºC for 4 h, resulting in a transparent sol. |
| 5 | The sol was statically placed at room temperature in an air atmosphere for 12 h to age. |
| 6 | The sol was put in a blast drying oven, and it was subjected to blast drying at 150 ºC for 5 h in an air atmosphere to form a gel. |
| 7 | The obtained gel was put in a muffle furnace and calcined for 3 h at 500 ºC in an air atmosphere; the calcination profile is shown in Figure S1. |
| 8 | The solid obtained after calcination was ground, tableted and sieved into particles of 40−60 mesh, which can be directly used for activity testing. |

https://doi.org/10.3390/xxxxx

## Section S2. Catalyst characterization

A series of characterization methods were employed to investigate the structure-efficiency relation of V-Pt-Ti catalysts. The details were as follow [1]: The textural properties of catalysts were evaluated by using a physisorption instrument (ASAP 2020 Plus, Micromeritics) at liquid nitrogen temperature (−196 ºC). The specific surface areas of catalysts were calculated by the $N_2$ adsorption−desorption isotherm using the Brunauer−Emmett−Teller (BET) equation. The average pore diameter, pore volume, and pore size distribution were calculated by the $N_2$ desorption isotherm using the Barrett−Joyner−Halenda (BJH) model.

The X-ray diffraction (XRD) tests of catalysts were carried out on an X-ray diffraction meter (Empyrean, PANalytical B.V., Almelo, Overijssel, Netherlands) using Cu K$\alpha$ ($\lambda$=0.154 nm) as a radiation source. The XRD diffractograms of catalysts were recorded in the 2θ range of 20–70 º with a scanning interval of 0.02 º.

The X-ray photoelectron spectra (XPS) of catalysts were measured by using a photoelectron spectrometer (AXIS-ULTRA DLD-600W, Shimadzu Corporation, Kyoto, Kyoto Prefecture, Japan) with Al K$\alpha$ as a radiation source. The binding energies of different elements were calibrated by C 1s peak at 284.6 eV.

The scanning electron microscope (SEM) images of catalysts were measured on a JSM-IT200 electron microscope (JEOL Ltd., Tokyo, Japan). The acceleration voltage was 200 kV.

The transmission electron microscopy (TEM) images of catalysts were measured on a JEM-2100F electron microscope (JEOL Ltd., Tokyo, Japan). The acceleration voltage was 200 kV, and the surface chemical analysis was conducted by using energy dispersive X-ray spectrometer (EDS).

$H_2$-temperature programmed reduction ($H_2$-TPR), $NH_3$-temperature programmed desorption ($NH_3$-TPD), and oxygen programmed desorption ($O_2$-TPD) were conducted on a chemisorption analyzer (Autochem II 2920, Micromeritics Instrument Corporation, Norcross, GA, USA). Before each $H_2$-TPR test, the sample was flushed in a He stream (50 mL/min) at 300 ºC for 30 min and then cooled down to 50 ºC. The reduction reaction was proceeded from 50 to 800 °C with a heating rate of 10 ºC/min. A mixture gas flow (50 mL/min) of 5 % $H_2$ and 95 % Ar was used as reducing gas.

For $NH_3$-TPD experiments, each sample was pretreated in a He stream (50 mL/min) for 30 min at 200 ºC and saturated with $NH_3$ at 50 ºC for 1 h. Then, the sample was treated by a He stream at 50 ºC for 1 h to eliminate physically absorbed $NH_3$. Finally, the sample was heated from 50 to 500 ºC at a ramping rate of 10 ºC/min in a He stream (50 mL/min), and $NH_3$-TPD data were recorded in the meantime.

For $O_2$-TPD experiments, each sample was pretreated in a He stream (50 mL/min) for 30 min at 200 ºC and saturated with $O_2$ at 50 ºC for 1 h. Then, the sample was treated by a He stream at 50 ºC for 1 h. Finally, the sample was heated from 100 to 900 ºC at a ramping rate of 10 ºC/min in a He stream (50 mL/min), and $O_2$-TPD data were recorded at the same time.

The in situ DRIFTS measurements were carried out on an FT-IR spectrometer (iS50, Thermo Fisher Scientific, Waltham, MA, USA) equipped with a reaction cell containing a KBr window (Praying Mantis, Harrick) and a program temperature controller. Typically, samples were pretreated in $N_2$ flow at 100 ºC for 30 min and then the background spectra were recorded. Subsequently, in situ DRIFTS spectra were recorded after the reaction gas was introduced into the reaction cell.

The in situ DRIFTS experimental processes of the reaction between $O_2$ and pre-adsorbed $NH_3$ were as follows: Firstly, catalysts were flushed by $N_2$ (100 mL/min) at 100 ºC. Then, catalysts were pre-adsorbed with 3000 ppm of $NH_3$ (100 mL/min) for 30 min for saturation and purged with $N_2$ (100 mL/min) for 30 min to eliminate physically adsorbed

$NH_3$. Next, 5 % $O_2$ (100 mL/min) was introduced into the reactor, and the IR spectra were recorded as a function of time.

## Section S3. $NH_3$-SCO performance comparison

The comparison for $NH_3$-SCO performance of the VPT-PC catalyst and other catalysts in previous work is shown in Table S6. $T_{100}$ represents the corresponding reaction temperature when the $NH_3$ removal efficiency was 100 %.

**Table S6.** $NH_3$-SCO performance comparison for the VPT-PC catalyst and other catalysts in previous work.

| Catalyst | Synthesis process | Experimental conditions | $T_{100}$ (°C) | $N_2$ selectivity at $T_{100}$ (%) | Ref. |
|---|---|---|---|---|---|
| VPT-PC | Precipitation | $NH_3$: 3000 ppm<br>$O_2$: 5 vol. %<br>$N_2$ as balance gas<br>GHSV: 60,000 $h^{-1}$ | 200 | 92 | **This work** |
| $V_{0.5}/Pt_{0.04}/TiO_2$ (0.04 wt.% Pt) | Two-step impregnation | $NH_3$: 5000 ppm<br>$O_2$: 10 vol. %<br>$N_2$ as balance gas<br>GHSV: 60,000 $h^{-1}$ | 250 | 77 | 1 |
| Pt/γ-$Al_2O_3$ (0.46% wt.% Pt) | Impregnation | $[NH_3]$ = 500 ppm,<br>$[O_2]$ = 5 vol. %, $N_2$ as balance gas<br>GHSV = 66,000 $h^{-1}$ | 250 | 50 | 2 |
| Pt/CeZr$O_x$ (1 wt.% Pt) | Impregnation | $[NH_3]$ = 200 ppm,<br>$[O_2]$ = 8 vol. %, $N_2$ as balance gas<br>GHSV = 100,000 $h^{-1}$ | 330 | 42 | 3 |
| Pt/Anatase-$TiO_2$ (0.1 wt.% Pt) | Wet impregnation | $NH_3$: 2000 ppm<br>$O_2$: 8 vol. %<br>$N_2$ as balance gas<br>GHSV: 60,000 $h^{-1}$ | 275 | 48 | 4 |
| Pt-ZSM-5 (2.55 wt.% Pt) | Ion-exchange | $[NH_3]$ = 1000 ppm,<br>$[O_2]$ = 4 vol. %, $N_2$ as balance gas<br>GHSV = 50,000 $h^{-1}$ | 200 | 71 | 5 |
| Pt-V-W/$TiO_2$ (0.1 wt.% Pt) | Wet impregnation | $NH_3$: 200 ppm<br>$O_2$: 5 vol. %<br>$N_2$ as balance gas<br>GHSV: 100,000 $h^{-1}$ | 250 | 50 | 6 |
| Pt-W/$ZrO_2$ (1.5 wt.% Pt) | Impregnation | $[NH_3]$ = 180 ppm,<br>$[O_2]$ = 8 vol. %, $N_2$ as balance gas<br>GHSV = 100,000 $h^{-1}$ | 300 | 58 | 7 |
| Ag/γ-$Al_2O_3$ | Wet impregnation | $[NH_3]$ = 500 ppm,<br>$[O_2]$ = 10 vol. %, Ar as balance gas<br>GHSV = 28,000 $h^{-1}$ | 180 | 83 | 8 |
| Ag/SiTi$O_x$ (10 wt.% Ag) | Impregnation | $[NH_3]$ = 500 ppm,<br>$[O_2]$ = 10 vol. %, $N_2$ as balance gas<br>GHSV = 28,000 $h^{-1}$ | 200 | 63 | 9 |
| CuFe$O_x$ | Sol-gel | $[NH_3]$ = 800 ppm,<br>$[O_2]$ = 3 vol. %, $N_2$ as balance gas<br>GHSV = 90,000 $h^{-1}$ | 250 | 91 | 10 |
| Cu$O_x$/γ-$Al_2O_3$ | Wet impregnation | $[NH_3]$ = 1000 ppm,<br>$[O_2]$ = 10 vol. %, $N_2$ as balance gas | 350 | 93 | 11 |

| | | | | | |
|---|---|---|---|---|---|
| | | GHSV = 50,000 $h^{-1}$ | | | |
| $FeTiO_x$ | Sol–gel | $[NH_3]$ = 1000 ppm, $[O_2]$ = 3 vol. %, $N_2$ as balance gas GHSV = 200,000 $h^{-1}$ | 400 | 91 | 12 |
| $Mn_2O_3$ | Thermal decomposition | $[NH_3]$ = 500 ppm, $[O_2]$ = 3 vol. %, He as balance GHSV = 20,000 $h^{-1}$ | 210 | 60 | 13 |

## Section S4. XRD quantitative phase analysis

According to previous studies [14-16], for a mixture containing two phases (phase A and phase B), the relationship between their mass fractions and diffraction peak intensities is as follows:

$$\frac{w_A}{w_B} = K_{A/B} \cdot \frac{I_A}{I_B} \quad \text{(S1)}$$

where

$w_A$ and $w_B$ are the mass fractions of phase A and phase B, respectively ($w_A + w_B = 1$);

$I_A$ and $I_B$ are the integrated intensities (or peak heights, which must be consistent) of the characteristic diffraction peaks of phase A and phase B, respectively;

$K_{A/B}$ is the relative reference intensity (dimensionless) of phase A to phase B, which can be retrieved from JCPDS cards [14-18].

$V_2O_5$ (JCPDS 19-1401) is defined as phase A and $TiO_2$ (ICDD 21-1272) as phase B, and the calculations were performed using Equation (S1). The results are shown in Table S7. It can be seen that the crystalline phases on the surfaces of both VPT-PC and VPT-SG catalysts are $TiO_2$, and their corresponding signal intensities are significantly lower than those of other Pt-V-Ti catalysts. This implies that VPT-PC and VPT-SG catalysts are largely amorphous.

**Table S7.** Synthetic procedures and technological parameters of the VPT-TD catalyst.

| Catalyst | Phase Name | Intensity | K Value | Mass Fraction (%) |
|---|---|---|---|---|
| VPT-IP | $V_2O_5$ | 156 | 1.2 | 1.7 |
| | $TiO_2$ | 10733 | | 98.3 |
| VPT-PC | $V_2O_5$ | 0 | | 0 |
| | $TiO_2$ | 2656 | | 100 |
| VPT-HD | $V_2O_5$ | 93 | | 1.3 |
| | $TiO_2$ | 8223 | | 98.7 |
| VPT-TD | $V_2O_5$ | 62 | | 0.8 |
| | $TiO_2$ | 9062 | | 99.2 |
| VPT-SG | $V_2O_5$ | 0 | | 0 |
| | $TiO_2$ | 8103 | | 100 |

## Section S5. TEM and EDS

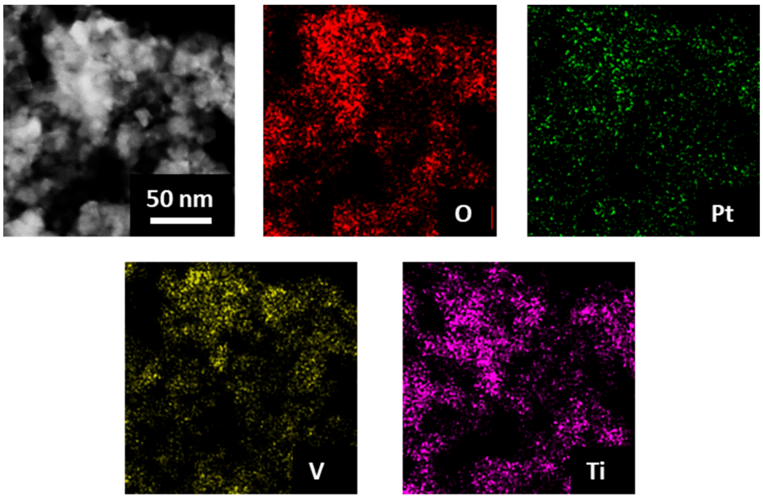


**Figure S2.** TEM and EDS image of the VPT-PC catalyst.

## Section S6. In situ DRIFTS

The Pt-Ti catalyst (denoted as PT), V-Ti catalyst (denoted as VT), and $TiO_2$ catalyst (denoted as T) were synthesized, and in situ DRIFTS tests were conducted on them to isolate the contributions of Pt, V, $O_\alpha$, and other factors. The synthesis processes of PT, VT, and T catalysts were mainly referenced to that of the VPT-PC catalyst, as shown in Tables S8–S10.

**Table S8.** Synthetic procedures and technological parameters of the PT catalyst.

| Steps | Contents |
|---|---|
| 1 | A $Pt(NO_3)_2$ solution (0.27 g) was added to 100 mL of deionized water. |
| 2 | The mixture was subjected to continuous stirring (200 rpm) at 60 °C for 30 min. |
| 3 | $TiO(SO_4)\cdot xH_2SO_4\cdot xH_2O$ (6 g) was added to the mixture. |
| 4 | $NH_4OH$ was added dropwise to the mixture obtained from previous step with continuous stirring (150 rpm) until the pH value of the mixture reached 10. |
| 5 | The obtained suspension solution was statically placed at room temperature in an air atmosphere for 12 h. |
| 6 | The precipitate was put in a blast drying oven, and blast drying was performed on it at 120 °C for 6 h in an air atmosphere. |
| 7 | The solid obtained after drying was put in a muffle furnace and calcined for 3 h in an air atmosphere; the calcination profile is shown in Figure S1. |
| 8 | The solid obtained after calcination was ground, tableted and sieved into particles of 40−60 mesh, which can be directly used for activity testing. |

**Table S9.** Synthetic procedures and technological parameters of the VT catalyst.

| Steps | Contents |
|---|---|

https://doi.org/10.3390/xxxxx

| | |
|---|---|
| 1 | $NH_4VO_3$ (1.46 g) was added to 100 mL of deionized water. |
| 2 | The mixture was subjected to continuous stirring (200 rpm) at 60 ºC for 30 min. |
| 3 | $TiO(SO_4)\cdot xH_2SO_4\cdot xH_2O$ (6 g) was added to the mixture. |
| 4 | $NH_4OH$ was added dropwise to the mixture obtained from previous step with continuous stirring (150 rpm) until the pH value of the mixture reached 10. |
| 5 | The obtained suspension solution was statically placed at room temperature in an air atmosphere for 12 h. |
| 6 | The precipitate was put in a blast drying oven, and blast drying was performed on it at 120 ºC for 6 h in an air atmosphere. |
| 7 | The solid obtained after drying was put in a muffle furnace and calcined for 3 h in an air atmosphere; the calcination profile is shown in Figure S1. |
| 8 | The solid obtained after calcination was ground, tableted and sieved into particles of 40−60 mesh, which can be directly used for activity testing. |

**Table S10.** Synthetic procedures and technological parameters of the T catalyst.

| Steps | Contents |
|---|---|
| 1 | $TiO(SO_4)\cdot xH_2SO_4\cdot xH_2O$ (6 g) was added to 100 mL of deionized water. |
| 2 | The mixture was subjected to continuous stirring (200 rpm) at 60 ºC for 30 min. |
| 3 | $NH_4OH$ was added dropwise to the mixture obtained from previous step with continuous stirring (150 rpm) until the pH value of the mixture reached 10. |
| 4 | The obtained suspension solution was statically placed at room temperature in an air atmosphere for 12 h. |
| 5 | The precipitate was put in a blast drying oven, and blast drying was performed on it at 120 ºC for 6 h in an air atmosphere. |
| 6 | The solid obtained after drying was put in a muffle furnace and calcined for 3 h in an air atmosphere; the calcination profile is shown in Figure S1. |
| 7 | The solid obtained after calcination was ground, tableted and sieved into particles of 40−60 mesh, which can be directly used for activity testing. |

The steps of in situ DRIFTS experiments were as follows: Initially, the catalysts were purged with $N_2$ at a flow rate of 100 mL/min at 200 ºC. Subsequently, the catalysts were subjected to the pre-adsorption of $NH_3$ with a concentration of 3000 ppm at 100 mL/min for 30 minutes to achieve saturation. After that, they were rinsed with $N_2$ (100 mL/min) for 30 minutes to remove physically adsorbed $NH_3$. Finally, 5 % $O_2$ (100 mL/min) was fed into the reactor, and IR spectra were recorded over time.

The in situ DRIFTS test results of PT, VT, and T catalysts are shown in Figure S3. After $O_2$ was introduced to feed gas, the intensity of bands that could be ascribed to $NH_3$

coordinated on Lewis acid sites (3383, 3386, 3380, and 3250 $cm^{-1}$), N-H stretching vibration modes of coordinated $NH_3$ species (3143, 3150, and 3139 $cm^{-1}$), bidentate nitrate species (1583 $cm^{-1}$), and $NH_4^+$ species coordinated at Brønsted acid sites (1427, 1425, and 1435 $cm^{-1}$) decreased gradually. The intensity of bands corresponding to bidentate nitrate species (1583, 1576, and 1582 $cm^{-1}$) increased gradually. This indicated that the $NH_3$ coordinated on Lewis acid sites, coordinated $NH_3$ species, and $NH_4^+$ species coordinated at Brønsted acid sites species on the surface of V-Pt-Ti catalysts could react with $O_2$, generating bidentate nitrate species.

As shown in Figure S3, when $O_2$ was introduced into the reaction cell, a clear peak corresponding to $NO_2$ species (1646 $cm^{-1}$) emerged in the in situ DRIFTS spectrum of the PT catalyst, which is consistent with the case of the VPT-PC catalyst. However, this peak was absent in the in situ DRIFTS spectra of other prepared catalysts under the same reaction conditions. This suggested that the presence of $NO_2$ species on the surface of the VPT-PC catalyst might be related to Pt species.

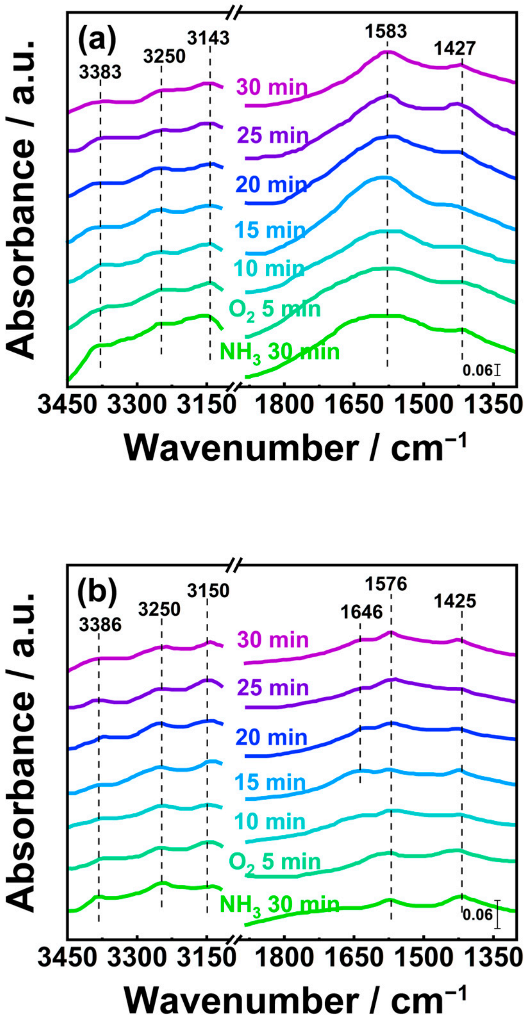


https://doi.org/10.3390/xxxxx

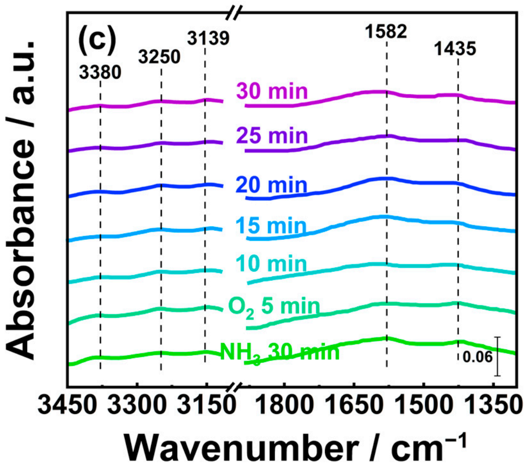


**Figure S3.** In situ DRIFT spectra of the VT (a), PT (b), and T (c) catalysts.

## Section S7. Long-term $N_2O$ emission

The VPT-PC catalyst with superior performance was selected as a research object, and the concentrations of $NH_3$, $N_2O$, NO, and $NO_2$ in the outlet gas during its 10 h continuous operation at 200 ºC were investigated. The results are presented in Figure S4. It could be seen that during 10 h continuous operation, $NH_3$ concentration in the outlet gas remained at 0 ppm, $N_2O$ concentration was ~50 ppm, NO concentration was ~200 ppm, and $NO_2$ concentration was 0 ppm. The concentration of $N_2O$ generated from the $NH_3$-SCO reaction over the VPT-PC catalyst was relatively low and remained stable during long-term operation.

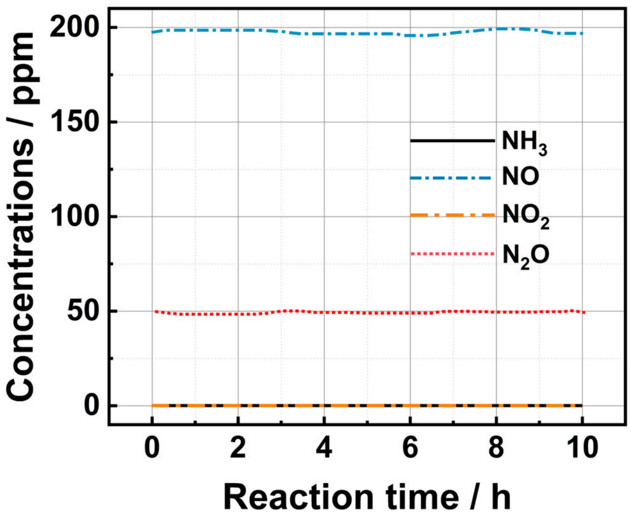


**Figure S4.** The stability test results of the VPT-PC catalyst. Reaction conditions: $[NH_3]in$ = 3000 ppm, $[O_2]in$ = 5 vol.%, GHSV = 60000 $h^{-1}$, T=200 ºC, and $N_2$ was the balance gas.

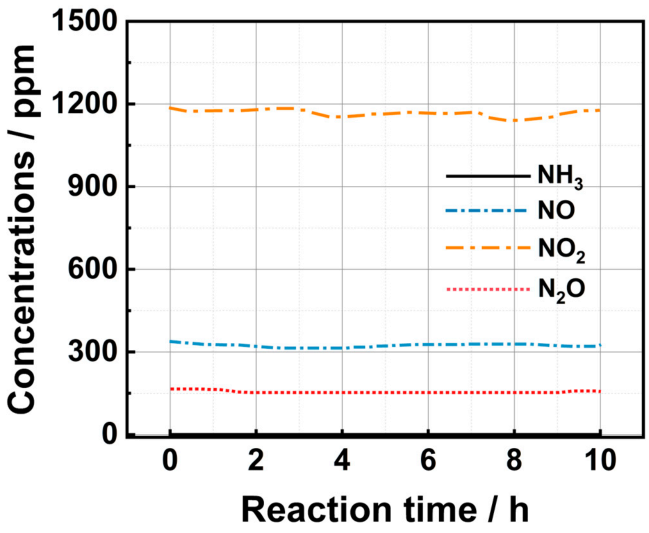


**Figure S5.** The stability test results of the PT catalyst. Reaction conditions: $[NH_3]in$ = 3000 ppm, $[O_2]in$ = 5 vol.%, GHSV = 60000 $h^{-1}$, T=200 ºC, and $N_2$ was the balance gas.

## Section S8. XPS data

**Table S11.** XPS results of V-Pt-Ti catalysts.

| **Sample** | **$V^{5+}/V^{3+}+V^{4+}+V^{5+}$ (%)** | **$O_\alpha/O_\alpha+O_\beta$ (%)** | **$Pt^0/Pt^0+Pt^{2+}$ (%)** |
|---|---|---|---|
| VPT-IP | 33.23 | 15.12 | 35.68 |
| VPT-PC | 37.39 | 15.26 | 38.32 |
| VPT-HD | 32.65 | 16.88 | 33.23 |
| VPT-TD | 29.83 | 15.02 | 33.02 |
| VPT-SG | 35.32 | 15.36 | 36.89 |

## Section S9. $H_2$-TPR data

**Table S12.** $H_2$ consumption values of V-Pt-Ti catalysts.

| **Sample** | **$H_2$ consumption value (mmol/g)** |
|---|---|
| VPT-IP | 0.73 |
| VPT-PC | 0.79 |
| VPT-HD | 0.66 |

https://doi.org/10.3390/xxxxx

| | |
|---|---|
| VPT-TD | 0.65 |
| VPT-SG | 0.69 |

## References


1. Gao, Y.; Han, Z.; Lu, S.; Pan, X. Influence of deposition order of dual active components on the $NH_3$-SCO performance of the bimetallic Pt-V system supported on $TiO_2$. *New J. Chem.* **2023**, *47*, 11143–11155.
2. Shrestha, S.; Harold, M.P.; Kamasamudram, K.; Yezerets, A. Selective oxidation of ammonia on mixed and dual-layer Fe-ZSM-5+Pt/$Al_2O_3$ monolithic catalysts. *Catal. Today* **2014**, *231*, 105–115. https://doi.org/10.1016/j.cattod.2014.01.024.
3. Sun, M.; Liu, J.; Song, C.; Ogata, Y.; Rao, H.; Zhao, X.; Xu, H.D.; Chen, Y. Different reaction mechanisms of ammonia oxidation reaction on Pt/$Al_2O_3$ and Pt/$CeZrO_2$ with various Pt states. *ACS Appl. Mater. Interfaces* **2019**, *11*, 23102–23111. https://doi.org/10.1021/acsami.9b02128.
4. Kim, G.J.; Kwon, D.W.; Shin, J.H.; Kim, K.W.; Hong, S.C. Influence of the addition of vanadium to Pt/$TiO_2$ catalyst on the selective catalytic oxidation of $NH_3$ to $N_2$. *Environ. Technol.* **2019**, *40*, 2588–2600. https://doi.org/10.1080/09593330.2018.1554004.
5. Sun, M.; Wang, S.; Li, Y.; Wang, Q.; Xu, H.; Chen, Y. Promotion of catalytic performance by adding Cu into Pt/ZSM-5 catalyst for selective catalytic oxidation of ammonia. *J. Taiwan Inst. Chem. Eng.* **2017**, *78*, 401–408. https://doi.org/10.1016/j.jtice.2017.06.045.
6. Byun, S.W.; Lee, S.J.; Kim, M.; Bae, W.B.; Shin, H.; Hazlett, M.J.; Kang, D.; Tesfaye, B.; Park, P.W.; Kang, S.B. High $N_2$ selectivity of Pt-VW/$TiO_2$ oxidation catalyst for simultaneous control of $NH_3$ and CO emissions. *Chem. Eng. J.* **2022**, *444*, 136517. https://doi.org/10.1016/j.cej.2022.136517.
7. Sun, M.; Wang, S.; Li, Y.; Xu, H.; Chen, Y. Promotion of catalytic performance by adding W into Pt/$ZrO_2$ catalyst for selective catalytic oxidation of ammonia. *Appl. Surf. Sci.* **2017**, *402*, 323–329. https://doi.org/10.1016/j.apsusc.2016.12.241.
8. Wang, F.; He, G.; Zhang, B.; Chen, M.; Chen, X.; Zhang, C.; He, H. Insights into the activation effect of $H_2$ pretreatment on Ag/$Al_2O_3$ catalyst for the selective oxidation of ammonia. *ACS Catal.* **2019**, *9*, 1437–1445. https://doi.org/10.1021/acscatal.8b03744.
9. Wang, F.; Ma, J.; He, G.; Chen, M.; Wang, S.; Zhang, C.; He, H. Synergistic effect of $TiO_2$-$SiO_2$ in Ag/Si-Ti catalyst for the selective catalytic oxidation of ammonia. *Ind. Eng. Chem. Res.* **2018**, *57*, 11903–11910. https://doi.org/10.1021/acs.iecr.8b02205.
10. Zhang, Q.; Wang, H.; Ning, P.; Song, Z.; Liu, X.; Duan, Y. In situ DRIFTS studies on CuO-$Fe_2O_3$ catalysts for low temperature selective catalytic oxidation of ammonia to nitrogen. *Appl. Surf. Sci.* **2017**, *419*, 733–743. https://doi.org/10.1016/j.apsusc.2017.05.056.
11. Gang, L.; Van Grondelle, J.; Anderson, B.G.; Van Santen, R.A. Selective low temperature $NH_3$ oxidation to $N_2$ on copper-based catalysts. *J. Catal.* **1999**, *186*, 100–109. https://doi.org/10.1006/jcat.1999.2524.
12. Long, R.Q.; Yang, R.T. Selective catalytic oxidation of ammonia to nitrogen over $Fe_2O_3$-$TiO_2$ prepared with a sol-gel method. *J. Catal.* **2002**, *207*, 158–165. https://doi.org/10.1006/jcat.2002.3545.
13. Lee, J.Y.; Kim, S.B.; Hong, S.C. Characterization and reactivity of natural manganese ore catalysts in the selective catalytic oxidation of ammonia to nitrogen. *Chemosphere* **2003**, *50*, 1115–1122. https://doi.org/10.1016/S0045-6535(02)00708-7.
14. Tamer, M. Quantitative Phase Analysis Based on Rietveld Structure Refinement for Carbonate Rocks. *J. Mod. Phys.* **2013**, *4*, 1169–1176. https://doi.org/10.4236/jmp.2013.48154.
15. Wang, Y.; Li, J.; Zhang, H. Quantitative Phase Analysis of Cement Clinker by Adiabatic XRD Method. *J. Mater. Res. Technol.* **2021**, *14*, 2165–2173. https://doi.org/10.1016/j.jmrt.2021.03.095.
16. Holý, V.; Chamard, V. High-Resolution X-Ray Diffraction Techniques for Crystalline Material Characterization. *J. Appl. Crystallogr.* **2017**, *50*, 555–560.
17. Bunaciu, A.A.; Aboul-Enein, H.Y. X-Ray Diffraction Instrumentation and Its Appl in Material Science. *Crit. Rev. Anal. Chem.* **2015**, *45*, 210–225. https://doi.org/10.1080/10408347.2014.962986.ication
18. Newsam, J.M.; Liang, K.S. Synchrotron X-ray diffraction studies of inorganic materials and heterogeneous catalysts. *Int. Rev. Phys. Chem.* **1989**, *8*, 289–338. https://doi.org/10.1080/01442358909353232.
